# A novel *MT-ATP6* variant associated with complicated ataxia in two unrelated Italian patients: case report and functional studies

**DOI:** 10.1186/s13023-024-03212-y

**Published:** 2024-05-16

**Authors:** Daniele Sala, Silvia Marchet, Lorenzo Nanetti, Andrea Legati, Caterina Mariotti, Eleonora Lamantea, Daniele Ghezzi, Alessia Catania, Costanza Lamperti

**Affiliations:** 1 Unit of Medical Genetics and Neurogenetics, Fondazione IRCCS Istituto Neurologico Carlo Besta, 20126 Milan, Italy; 2 Department of Pathophysiology and Transplantation (DEPT), University of Milan, 20122 Milan, Italy

**Keywords:** *MT-ATP6*, Ataxia, ATP synthase, Mitochondria, OXPHOS, Complex V, Cybrids, Oxygen consumption

## Abstract

**Background:**

*MT-ATP6* is a mitochondrial gene which encodes for the intramembrane subunit 6 (or A) of the mitochondrial ATP synthase, also known asl complex V, which is involved in the last step of oxidative phosphorylation to produce cellular ATP through aerobic metabolism. Although classically associated with the NARP syndrome, recent evidence highlights an important role of *MT-ATP6* pathogenic variants in complicated adult-onset ataxias.

**Methods:**

We describe two unrelated patients with adult-onset cerebellar ataxia associated with severe optic atrophy and mild cognitive impairment. Whole mitochondrial DNA sequencing was performed in both patients. We employed patients’ primary fibroblasts and cytoplasmic hybrids (cybrids), generated from patients-derived cells, to assess the activity of respiratory chain complexes, oxygen consumption rate (OCR), ATP production and mitochondrial membrane potential.

**Results:**

In both patients, we identified the same novel m.8777 T > C variant in *MT-ATP6* with variable heteroplasmy level in different tissues. We identifed an additional heteroplasmic novel variant in *MT-ATP6*, m.8879G > T, in the patients with the most severe phenotype. A significant reduction in complex V activity, OCR and ATP production was observed in cybrid clones homoplasmic for the m.8777 T > C variant, while no functional defect was detected in m.8879G > T homoplasmic clones. In addition, fibroblasts with high heteroplasmic levelsof m.8777 T > C variant showed hyperpolarization of mitochondrial membranes.

**Conclusions:**

We describe a novel pathogenic mtDNA variant in *MT-ATP6* associated with adult-onset ataxia, reinforcing the value of mtDNA screening within the diagnostic workflow of selected patients with late onset ataxias.

**Supplementary Information:**

The online version contains supplementary material available at 10.1186/s13023-024-03212-y.

## Background

The key function of mitochondria is the generation of the main source of cellular energy in the form of ATP (adenosine triphosphate), through the activity of the respiratory chain (RC). RC is located within the inner mitochondrial membrane and consists of four multiprotein complexes (complexes I-IV) which act as electron transporters generating a proton electrochemical gradient, essential for the mitochondrial ATP synthase (complex V, cV) to produce ATP from adenosine diphosphate and inorganic phosphate.

Most of the mitochondrial diseases are caused by mutations in nuclear or mitochondrial DNA affecting components and regulatory factors of the RC, leading to a predominant dysfunction of the oxidative phosphorylation (OXPHOS) system [23]. The *MT-ATP6* gene encodes for the ATP synthase subunit A of cV, which is an essential constituent of the proton pore that drives ATP production and is also required for the accurate assembly of cV. Maternally inherited mutations of this subunit potentially result in a disruption of enzyme catalytic activity and represent the most common genetic cause of complex V deficiency (OMIM *516,060), [2].

Classic syndromic phenotypes associated with *MT-ATP6* mutations are Leigh syndrome (OMIM #256,000), NARP (neuropathy, ataxia and retinitis pigmentosa; OMIM #551,500) and hypertrophic cardiomyopathy [22].

Since the appearance of the first reports describing phenotypes associated with the most prevalent *MT-ATP6* genetic variant (i.e., m.8993 T > G), over 200 patients have been reported in literature, showing a wide clinical heterogeneity, even amongst family members carrying the same mutation, and different effects on mitochondrial functional essays [7].

Within the last ten years a growing number of adult onset spinocerebellar ataxia (SCA) cases have been reported in association with *MT-ATP6* variants; most cases exhibit complex phenotypes with a variable combination of neuropathy, spasticity, renal disease, psychiatric or cognitive dysfunction, and diabetes, but some of the phenotypes described were indistinguishable from the most known SCA due to nuclear mutations [16].

In this article, we report two patients from unrelated families affected by a complex and unique adult onset SCA phenotype and harboring the same novel missense variant in *MT-ATP6*. The patient showing the most severe clinical phenotype was also found to carry an additional missense variant within the same gene. We performed in silico analysis and functional studies on patients’ fibroblasts and cybrids to validate the pathogenicity of the identified variants.

## Materials and methods

### Muscle biopsy

Left quadriceps muscle biopsy was obtained from patient 1 after informed consent, frozen in isopentane and stained for histology as described in [19].

### Primary cell cultures and cybrids generation

Primary fibroblast lines were obtained from skin biopsies of both patients and their mothers and cultured according to the methods reported in [15].

Cybrid cell lines were generated using donor mitochondria from patients’ enucleated fibroblasts fused with Rho-0 cell lines treated with ethidium bromide. The whole procedure of cybrids generation and culturing is described in [4]. Purity of cybrid lines was evaluated with D1S80 microsatellite amplification assay.

### mtDNA sequence analysis and evaluation of mtDNA variant heteroplasmy

The whole mtDNA was PCR-amplified and then sequenced by an NGS approach on MiSeq Illumina platform as described in [12].

To quantify the heteroplasmy of generated clones, we employed two different methods. Firstly, a PCR Restriction Fragment Length Polymorphism (RFLP) assay was set up using *Alu*I restriction enzyme (New England Biolabs) for the m.8777 T > C variant. The method was applied to PCR amplicons spanning from position m.8727 to m.8850 (reverse primer 1). Notably the forward primer (Metabion International AG) contained a modified nucleotide C > G corresponding to position m.8775 of *MT-ATP6* gene; this is essential to introduce a palindromic consensus sequence (AGCT) for the digestion by *Alu*I, which is enabled to cut the wild type (wt) fragments, leaving uncut the fragments containing the nucleotide change m.8777 T > C. Upon overnight incubation with *Alu*I, DNA amplicons were separated on 8% acrylamide gel to evaluate the ratio between mutated/wt mtDNA in each cybrid clone.

Modified forward primer (m.8727–8776), with the modified nucleotide in bold:

5’-CTGATCTCTTATACTAGTATCCTTAATCATTTTTATTGCCACAACTAA**G**C-3'

Reverse primers utilized:

- 1) 5’-TAAGGGGATGGCCATGGCTA-3' (m.8850–8840).
- 2) 5’-GATAAGGGGTGTAGGTGTGC-3' (m.8940–8930).

The RFLP method provided an estimation of the heteroplasmy level for each clone; thereafter, clones of interest were selected and their percentage of heteroplasmy validated through an NGS approach. PCR fragments spanning from m.8727 to m.8940 (reverse primer 2) were processed with Nextera XT DNA Library Preparation kit (Illumina) and sequenced afterwards by MiSeq Illumina. This method allowed greater accuracy in heteroplasmy quantification. Furthermore, it provided the possibility to quantify also the heteroplasmy of the second variant (m.8879G > T) in clones derived from patient 1. High and low heteroplasmic clones were selected from both patients, as well as clones with different combinations of the two genetic variants present in patient 1. Selected cybrid lines were subsequently cultured to be used for the following experimental tests.

### Biochemistry of respiratory chain

Biochemical assays to evaluate all RC complex activities were performed on both patients’ fibroblasts and derived cybrids, as already shown in [1, 18].

### Fluorescence imaging

Primary fibroblasts were analysed through live fluorescence imaging using tetramethylrhodamine methyl ester (TMRM) staining, to study mitochondrial membrane potential (MMP). TMRM is a fluorescent dye able to accumulate in functional mitochondria with intact membrane potential, allowing a quantitative assay of MMP [8]. Fibroblasts were cultured in glass-bottom dishes (Ibidi) with fibroblasts’ growth medium [15], washed with PBS and incubated for 30 min at 37 °C with medium containing 20 nM TMRM stock solution (Invitrogen) and additional 5 µM Hoechst dye (Invitrogen), to simultaneously stain nuclei. A control dish was prepared using the same staining medium supplemented with 0.2 µM valinomycin (uncoupling agent to disrupt MMP) as positive control of the experiment. After incubation, medium was removed, cells washed with PBS and images acquired through fluorescent confocal microscope, at 574 nm emission wavelength. Hyper-stacked images were analysed through ImageJ software in order to calculate the Corrected Total Cell Fluorescence (CTCF) normalised on cell area, using the following formula:

$$[{\text{CTCF}}/\mathrm{cell\,area}=(\mathrm{Integrated\,Density}-(\mathrm{Area\,of\,selected\,cell}*\mathrm{Mean\,Background\,Fluorescence}))/\mathrm{Area\,of\,selected\,cell}].$$

### Seahorse

Oxygen Consumption Rate (OCR) was assessed through Seahorse XF96 technology, following the protocol described in [10]. This method also allows to infer ATP production.

### Statistical analysis

Statistical analyses were performed through Prism5 software. Data were tested for normality of distribution (Shapiro–Wilk test) and analysed using statistical tests ANOVA (for normal distributions) and Kruskall-Wallis (for non-normal distributions), respectively followed by Tukey’s honestly significant difference (HSD) and Dunn’s Multiple Comparisons tests. Statistically significant differences are shown in the figures associated to the underlying bars; p-value legends and statistical tests utilized are reported in the captions.

## Results

### Patients’ clinical description

#### Patient 1

The first patient is a 43 years old man born from healthy unrelated parents, after a pregnancy complicated by oligohydramnios. Two febrile seizures were reported in childhood. Psychomotor development was normal until the second infancy, when he started manifesting cognitive impairment and gait unsteadiness. Starting early during development, nocturnal muscle cramps and calf muscle pain at rest were also reported with elevated creatine phosphokinase (CPK). Since he was 10 years old, he complained recurring headache episodes in the left fronto-orbital region accompanied by emesis and nausea. At the age of 21 he was diagnosed with nephrotic syndrome with IgA mesangial deposits. Over time, he also exhibited progressive neurosensorial deafness that required the use of hearing aid, bilateral cataract and retinal dystrophy, hypogonadotropic hypogonadism, hypertension, bowel dysmotility with alternating constipation and diarrhoea; insulin-dependent diabetes was noted when he was 38.

The patient came to our Institute at 40 years of age. Family history was negative for neurological diseases. General observation showed the presence of short stature and mild dysmorphic features. Neurological exam revealed multidomain neuropsychological defects, severe visual impairment, ataxic stance and gait that required walking aids. Segmentary coordination was altered in lower limbs, and mild defective diadochokinetic was also detected. Hypotrophic muscles and pes cavus with Achilles tendon retraction were present. Muscle strength was only slightly impaired on tight and feet dorsal flexion. Clear pyramidal signs (mild hypertonia, bilateral extensor plantar responses and brisk deep tendon reflexed) were described on lower limbs.

Blood tests showed poor control of diabetes, slightly increased muscle enzymes (CPK 386 U/I—normal range 24–195) and IgA levels. Selective glomerular proteinuria was detected by urine analysis.

**Fig. 1:**
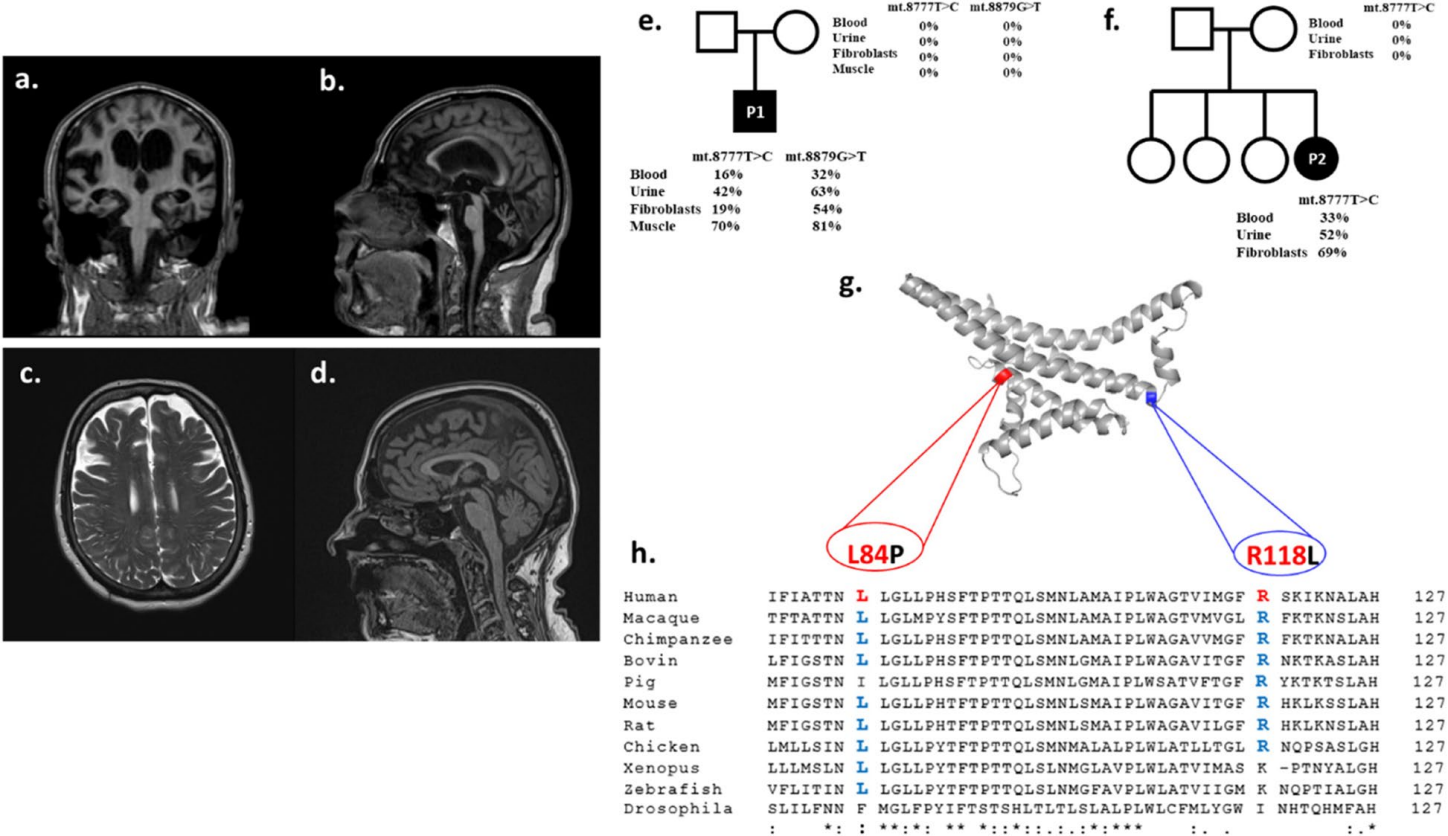
Brain MRI of patient 1 (**a-b**) shows a severe atrophy of cerebellum, brainstem and spinal cord (sagittal FLAIR weighted brain MRI on the left) and widespread cortical and hippocampal gray matter loss with ventricular enlargement (coronal FLAIR weighted brain MRI on the right). Brain MRI of patient 2 (**c-d**) shows frontal lobe sulci enlargement (axial T2 weighted brain MRI on the left) and cerebellar atrophy (coronal FLAIR weighted brain MRI on the right). Pedigrees of patient 1 (**e**) and patient 2 (**f**), showing the heteroplasmy level of the identified *MT-ATP6* variants in all analyzed tissues. 3D model of human ATP synthase subunit a (**g**) showing the location of the identified variants and corresponding phylogenetic analysis of amino acid conservation (**h**)

Brain MRI showed a severe atrophy of cortical regions, hippocampus, brainstem and cervical spinal cord with enlarged ventricles and sulci, except for the higher parasagittal region. Moreover, a severe global cerebellar atrophy was evident with minimal cortical hyperintensity and deep grey matter calcifications. Deep white matter presented confluent T2 signal hyperintensities mainly attributable to chronic hypoxia but also to minimal transependimal resorption (Fig. 1-a; b). Spectroscopy resulted in normal distribution of metabolite peaks. Multimodal evoked potentials showed global central system dysfunction, with a greater involvement of visual and auditory systems. Electromyography and conduction studies were suggestive for a severe mixed sensory-motor axonal polyneuropathy.

The patient underwent muscle biopsy that showed normal distribution of oxidative enzymes, predominance of clustered type I fibres and smaller clusters of type II fibres. Overall, hystoenzymatic analysis suggested a neurogenic damage without signs of mitochondrial dysfunction. The patient’s DNA was screened by a multigene panel for spinocerebellar ataxias, without any abnormal finding.

#### Patient 2

Patient 2 is a 47 years old lady. Born from unrelated parents, she was one of 4 sisters and worked as a kindergarten teacher. She was evaluated for the first time in our Institute when she was 45. Family history was negative for neurological diseases. In her forties she became aware of "blurred vision" and reduced visual acuity. From then on, she manifested walking imbalance initially attributed to vision disturbance, frequent cramps on lower limbs and urinary urgency. Ophthalmological evaluation at 42 years diagnosed severe bilateral visual loss (1/10), possibly attributable to retinal dystrophy. During the first hospitalization a brain MRI was performed, showing atrophic cerebellar cortex and peduncles (Fig. 1-c; d). Photoreceptor dysfunction was detected by electroretinography (ERG), with increased latency of Visual Evoked Potentials (VEPs); electromyography (EMG) was consistent with a severe axonal sensory-motor polyneuropathy.

Levels of phytanic and pristanic acid, cholestanol, alpha-fetoprotein were within normal ranges and anti-cerebellar antibodies were absent. CPK were within normal range. (109 U/L). Genetic testing ruled out abnormal repeat expansion in SCA1-2–3-6–7-17 and Friedreich ataxia genes; also, the patient was negative for common mtDNA mutations associated with NARP.

A comprehensive neuropsychological assessment documented defective short-term memory skills, executive functionand attention. Neurological examination showed oculomotor apraxia, segmentary incoordination and severely ataxic stance and gait, with broad swinging, truncal forward flexion and need for walking aid Strength evaluation showed a mild weakness of girdles and a diffuse and severe proximo-distal weakness on lower limbs associated with mild pyramidal hypertonia. Assessment of praxis, diadochokinesis and language articulation also showed mild functional impairment. Her neurological conditions slowly worsened overtime, with the appearance of dysphagia, restrictive respiratory insufficiency requiring non-invasive ventilation (NIV) and neck muscles weakness.

### Molecular analyses

The entire mtDNA was sequenced in DNA extracted from biopsy muscle of patient 1 identifying two different nucleotide changes on the *MT-ATP6* gene: m.8777 T > C and m.8879G > T, causing the amino acid substitutions p.Leu84Pro and p.Arg118Leu, respectively (Fig. 1-g). Both variants were absent in online databases (Mitomap, gnomAD 3.1 and Helix Mitochondrial database). Their heteroplasmy level was high in muscle (70% and 81%, respectively) and was also evaluated on blood, fibroblasts and urine sediment (Fig. 1-e).

Whole mtDNA sequencing was performed on DNA from blood of patient 2, showing the presence of the m.8777 T > C variant in *MT-ATP6*. Heteroplasmy was then tested in different specimens, and resulted 33% on blood, 52% on urinary sediment, 69% on cultured fibroblast (Fig. 1-f).

Both variants identified in this study were alleged de novo, as they were not found in blood, urine and fibroblasts from patients’ respective mothers*. *In silico analysis showed a high score of phylogenetic conservation for both variants (Fig. 1-h) and several prediction tools predicted them to be deleterious (Table 1). In silico mutagenesis using DynaMut software predicted p.Leu84Pro variant to be destabilizing on subunit A of ATP synthase 3D structure due to a gain in flexibility upon leucine to proline substitution. Also, p.Arg118Leu is predicted to affect protein function, due to substitution of a polar acidic aminoacid with a nonpolar one.

**Table 1:** In silico prediction of variants pathogenicity

	**PolyPhen2**	**SIFT**	**PROVEAN**	**Meta SNP**	**MITOMASTER**
Variant	Prediction (score^a^)	Prediction (score^b^)	Prediction (score^c^)	Prediction (score^a^)	Conservation score
m.8777 T > C / p.Leu84Pro	Probably damaging (1)	Deleterious (0)	Deleterious (-5.95)	Disease (0.76)	91.1%
m.8879G > T / p.Arg118Leu	Probably damaging (0.99)	Neutral (0.66)	Deleterious (-6.32)	Disease (0.54)	95.5%

^a^ minimal score 0, maximum 1

^b^ minimal score 1, maximum 0

^c^ minimal score -14, maximum 14

### Functional validation

**Fig. 2:**
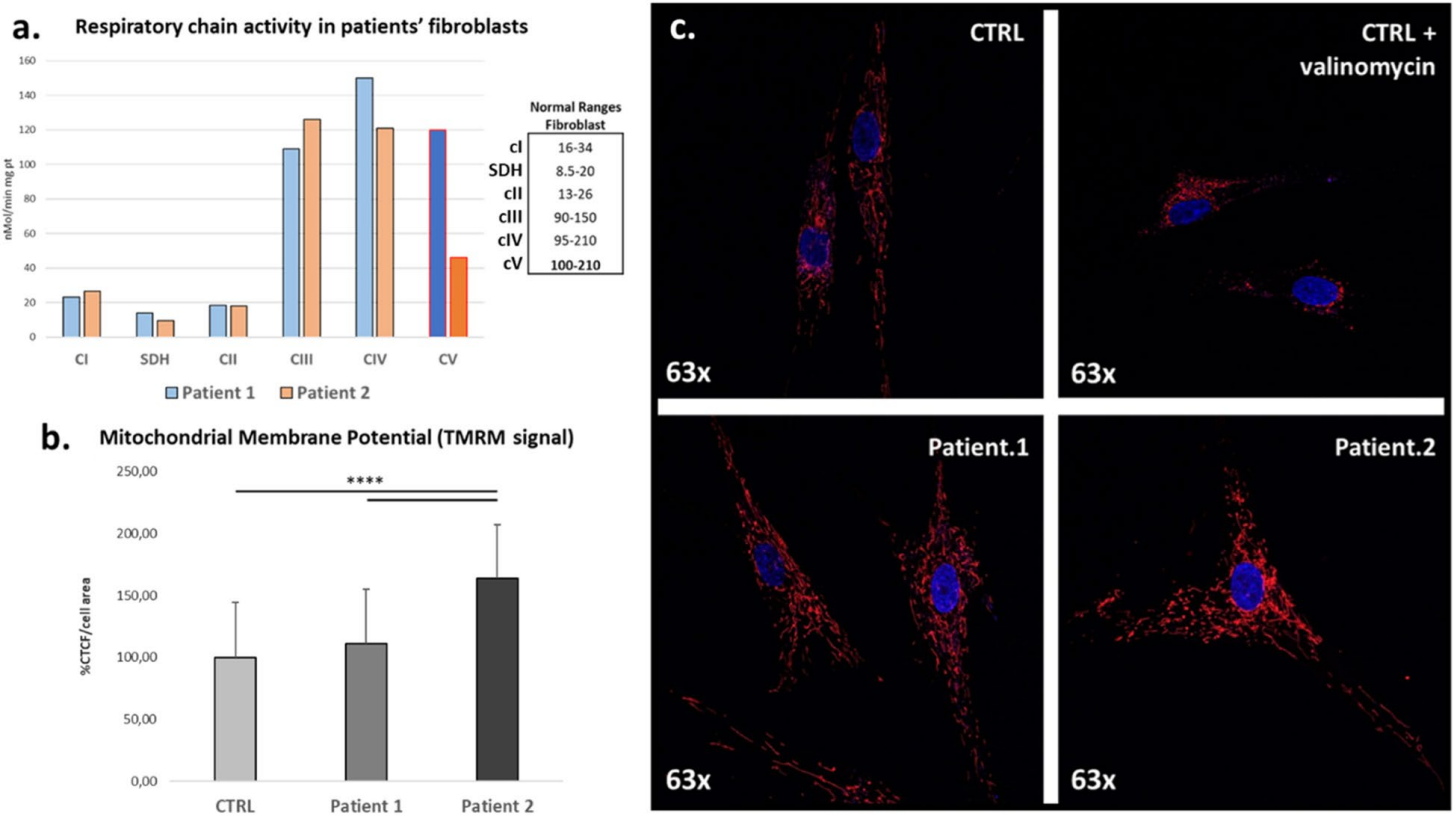
**a** Activity of the respiratory chain complexes measured in patients’ primary fibroblast lines, with relative normal ranges reported in the right table; cV activity is shown in darker colours. **b** Comparative analyses of TMRM signal expressed in CTCF/cell area [CTCF = Integrated Density – (Background Mean Fluorescence * Cell Area)], calculated as percentage of mean control signal of two experiments. **c** Representative panel of TMRM experimental conditions, respectively: control fibroblasts, control treated with valinomycin and both patients’ fibroblasts. Red signal intensity estimates MMP of functional mitochondria; nuclei are stained with Hoechst and visualized in blue. Statistical tests Kruskal–Wallis and Dunn’s Multiple Comparisons were performed to calculate p-values [****p < 0,0001; ***p < 0,001; **p < 0,01; *p < 0,05]

Primary fibroblasts were obtained from skin biopsies of both patients. Heteroplasmy levels of *MT-ATP6* variants were quantified through NGS approach and are reported in Fig. 1 (panels e–f). First, the activity of all respiratory chain complexes (cI-cV) was measured on digitonized pellets of both fibroblast lines. While patient 2 displayed an isolated cV deficiency (with residual activity of 45nMol/min normalized to citrate synthase activity and total protein amount; reference range of 100-210nMol/min), no defect was detected in patient 1 fibroblasts (Fig. 2-a). These data are consistent with the corresponding m.8777 T > C heteroplasmy levels, which is 69% in patient 2 fibroblasts, but only 19% in patient 1 cells.

Subsequently, confocal microscopy was used to estimate the MMP on live fibroblasts stained with TMRM. We observed a significantly higher mean MMP in patient 2 fibroblasts, compared to both control and patient 1 cells (Fig. 2–b; c). Again, this finding correlates with the higher m.8777 T > C heteroplasmy level in patient 2 fibroblasts.

Cybrid lines with different heteroplasmy levels of m.8777 T > C variant were generated from patient-derived cytoplasts on a 143b Rho-0 nuclear background. Using PCR–RFLP with *Alu*I restriction enzyme, we evaluated heteroplasmy levels to select clones with different m.8777 T > C heteroplasmic conditions: wild type (0%), intermediate heteroplasmy level (in a range spanning between 40 and 70%) and nearly homoplasmic (90–100%).

**Fig. 3:**
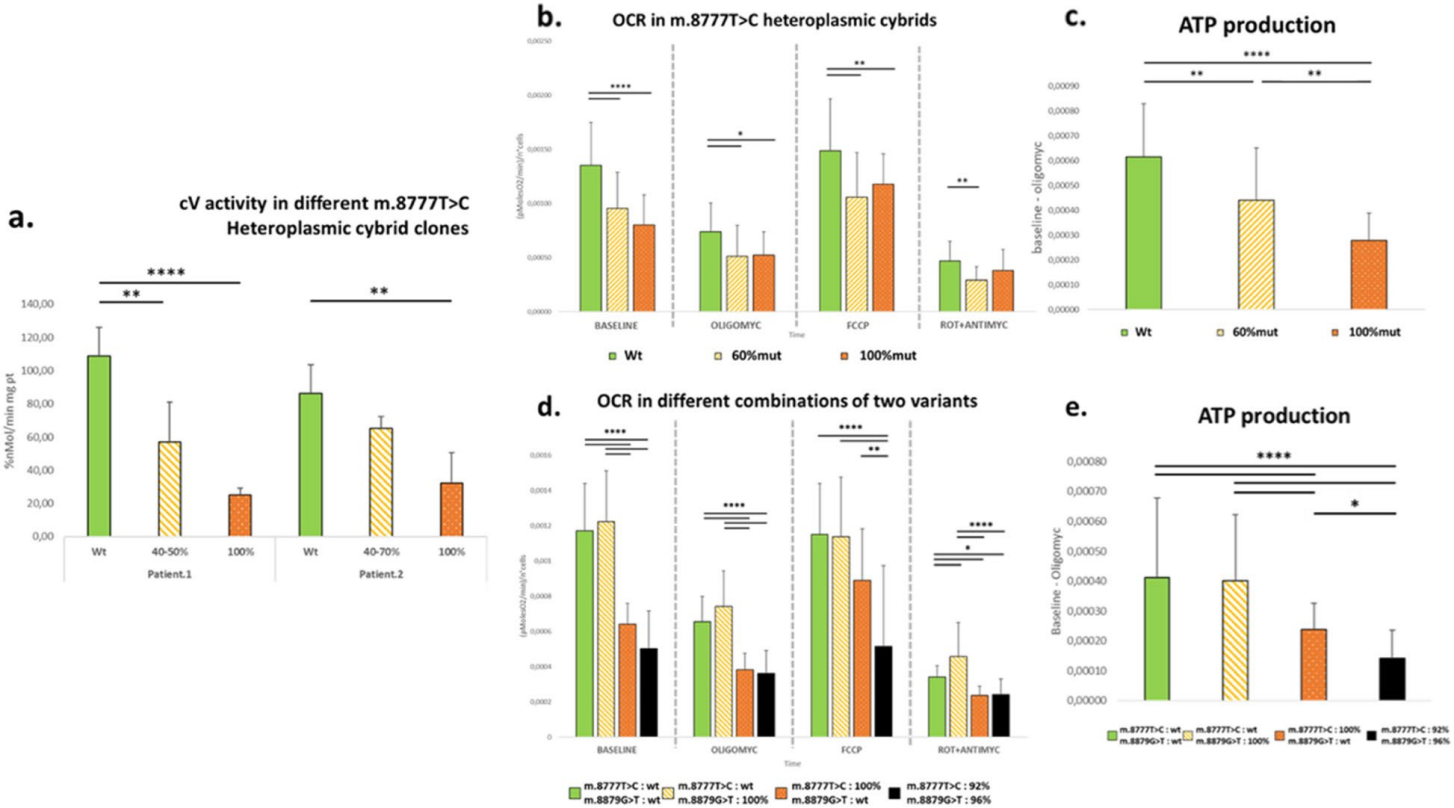
**a** CV activity compared in multiple cybrid clones derived from patient 1 and patient 2: wt (0%), intermediate heteroplasmy (40–70% range) and homoplasmy (100%) of m.8777 T > C variant. **b** Seahorse-XF OCR assessment performed on three cybrid cell lines derived from patient 2, carrying three different m.8777 T > C heteroplasmy levels (0%, 60% and 100%). **d** OCR pattern of Seahorse-XF experiment performed on selected cybrid clones derived from both patients, with different combinations heteroplasmy for m.8777 T > C and m.8879G > T variants, validated through NGS approach. Graphs (**c**) and (**e**) report the indirect measurement of ATP production calculated through the equation [Baseline OCR – Oligomycin OCR], from the respective Seahorse-XF experiments (**b**, **d**). Statistical analyses ANOVA and post hoc Tukey’s HSD tests were performed to calculate p-values [****p < 0,0001; *** p < 0,001; ** p < 0,01; * p < 0,05]

The activity of respiratory chain complexes was evaluated in cybrid clones representative for the three heteroplasmic conditions. No reduction was observed in the activity of cI, cII, cIII and cIV for all clones, while cV activity exhibited a noticeable reduction compared to the corresponding wt clones, directly related to the ascending heteroplasmy of m.8777 T > C variant (data not shown). Biochemical analyses of cV activity were replicated with additional generation of cybrid clones carrying wt, intermediate heteroplasmic or homoplasmic m.8777 T > C variants. In both patients-derived lines, a significant defect in cV activity was revealed in clones carrying homoplasmic m.8777 T > C variant, with evident trend of decreasing cV activities measured in the heteroplasmy conditions (40–70%) compared to the wt (Fig. 3-a).

OCR analyses were performed on the same patients’ cybrid lines (Fig. 3-b), revealing a reduced aerobic metabolism in cybrid clones with higher m.8777 T > C heteroplasmy levels, particularly noticeable in baseline OCR. Treatment with oligomycin – a specific cV inhibitor – allows to indirectly infer the ATP production of the studied cell lines [10]. Thus, we calculated the difference between baseline OCR and OCR after oligomycin administration in our cybrid clones, observing a significant reduction in ATP production, proportional to the m.8777 T > C heteroplasmy level (Fig. 3-c).

Considering the greater clinical severity of patient 1, we also investigated the possible contribution of the m.8879G > T variant to the biochemical defect. Heteroplasmy levels of both variants were assessed through NGS in cybrid clones derived from patient 1. Different combinations of wt and variable heteroplasmy of m.8777 T > C and m.8879G > T variants were tested for the specific biochemical activity of cV and OCR capability. While a defective respiration was confirmed in cybrids homoplasmic for m.8777 T > C, we did not detect any significant functional impairment in homoplasmic m.8879G > T cybrids (Fig. 3-d). Concomitant expression of very high percentage of both variants did not cause any significant change in overall oxidative capacity compared to homoplasmic m.8777 T > C clones. However, a slight decrease of maximal respiration after FCCP injection was observed in clones harbouring both heteroplasmic (> 90%) variants compared to homoplasmic m.8777 T > C clones. Comparable results were obtained by indirect calculation of ATP production, which was significantly defective just in cybrid lines carrying homoplasmic m.8777 T > C variant (Fig. 3-e) although a slightly significant further reduction is noticeable in cybrids carrying both variants. The latter findings may suggest a possible deleterious contribution of m.8879G > T if coexistent with m.8777 T > C.

## Discussion

The complexity of genotype–phenotype correlation with a high clinical variability (even within the same families and/or similar heteroplasmy levels) and the frequent lack of specificity of muscle histology and clinical tests make the identification of a mitochondrial disease particularly puzzling [2, 5, 17]. In the present study, we identified a novel *MT-ATP6* variant (m.8777 T > C) in two unrelated patients presenting with an overlapping clinical phenotype dominated by adult-onset cerebellar ataxia, which comprises also severe visual and cognitive impairment. *MT-ATP6* pathogenic variants have been linked to a broad variety of phenotypes besides the most known NARP and Leigh syndromes, including cases of spinocerebellar ataxia (SCA). Most of them presented with a complex combination of symptoms oftentimes including peripheral neuropathy, diabetes and ophthalmological abnormalities suggestive for a mitochondrial disease. However, patients with isolated ataxia, clinically indistinguishable from SCA caused by nuclear DNA mutations, have also been described [16].

Previous functional studies revealed how known *MT-ATP6* variants baffle the intra-enzymatic coupling between proton transport and the rotor mechanics of ATP synthase (cV) [9]. However, the observed downstream molecular defects exhibit a broad mutation-specific heterogeneity: most common findings include reduced cV activity with subsequent deficit of ATP production, altered sensitivity to oligomycin, ROS overproduction and abnormalities in MMP [11]. Indeed, different *MT-ATP6* mutations were found to either hinder proton transit, inducing proton accumulation in intermembrane space and subsequent hyperpolarization of mitochondrial membrane, or, conversely, to trigger a proton leak effect, which leads to membrane potential loss [7]. In addition, since the subunit 6 of cV plays an important role in oligo-/dimerization of ATP-synthases, some variants were found to interfere with the cV holocomplex assembly [3, 11]. It was reported that the main common molecular effect observed in samples carrying highly heteroplasmic *MT-ATP6* pathogenic variants is a reduced basal level of oxygen consumption. Since each variant leads to a subset of the above mentioned molecular and cellular dysregulations, functional validation is often complicated and requires multiple functional approaches [7].

The m.8777 T > C variant was assumed de novo in both patients and was predicted pathogenic by in silico analyses. Patient 2 fibroblasts, carrying high level of m.8777 T > C heteroplasmy (69%), showed a significant cV activity reduction and the hyperpolarization of mitochondrial membranes, while no such alterations were observed in patient 1 fibroblasts with low heteroplasmy (19%). These results provided evidence for pathogenicity of m.8777 T > C, also allowing to hypothesize a clogging effect on ATP synthase proton transport activity.

The generation of cybrid clones with different heteroplasmy levels on a homogeneous nuclear background confirmed that high heteroplasmy levels of m.8777 T > C variant lead to reduced cV activity, cellular OCR and ATP production, thus supporting the evidence of pathogenicity predicted by in silico tools and results on fibroblasts.

Patient 1, overall presenting with a more severe clinical presentation, was also found to harbour a second de novo variant in *MT-ATP6* (m.8879G > T). Cybrid studies performed on clones harbouring only this variant showed no significant functional defect. Based on these findings, a major role of m.8879G > T as pathogenic variant can be excluded. We also tested the co-presence of both variants, but this condition did not markedly worsen the functional impairment caused by the pathogenic m.8777 T > C variant alone. However, some parameters were partly affected indicating a possible role as genetic modifier for the m.8879G > T variant if coexistent with m.8777 T > C.

To possibly explain the significant difference in clinical severity between the two patients we also have to consider a few subject-specific drivers: first of all, heteroplasmy levels are not evenly distributed across tissues and could exhibit a great variability; besides, it is possible that additional modifier genes within the unique genetic (either mitochondrial or nuclear) background of each patient could influence the phenotypic expression of the identified variants.

We also need to consider the intrinsic limitations of cybrids as a model for mitochondrial diseases: having a tumour derivation, cybrids are predominantly based on glycolytic metabolism, which is in contrast with the highly oxidative metabolism of tissues typically affected in mitochondriopathies. Together with the unpredictable heteroplasmy shifts and presence of mixed population within the same cybrid line, the maintenance of the experimental di-genomic condition is not always fully reliable, especially when cells are kept in culture for long periods [13, 21]. To avoid this last intrinsic issue, heteroplasmy of cybrid samples was always tested in parallel for each experiment.

Several rescuing strategies have been proposed for *MT-ATP6* genetic defects, sometimes showing good preliminary results in vitro. These includes pharmacological treatments such as α-ketoglutarate and aspartate supplementation, or molecular therapies inducing allotopic or even xenotopic expression of functional *MT-ATP6* gene [6, 11, 20]. A recent study proposed phosphodiesterase-5 (PDE5) inhibitors (Sildenafil and analogues) as possible therapeutic agents for those mtDNA defects leading to increased MMP, based on the beneficial effects observed in vitro on iPS-derived neural progenitors harbouring a known *MT-ATP6* mutation [14]. Because of the hyperpolarization observed in fibroblasts carrying the m.8777 T > C variant, this pharmacological approach could be suitable for the cases presented here. Personalized medicine in the field of ATP-synthase pathology represents a promising future perspective warranting further investigation to be translated on patients.

## Conclusions

In conclusion, our results show that the novel m.8777 T > C variant in *MT-ATP6* is pathogenic at multiple molecular levels, primarily impairing cV activity, ATP production, aerobic metabolism and MMP.

Our report represents a further confirmation about the important role of the *MT-ATP6* gene within the landscape of genetic ataxias, adding evidence for a selective vulnerability of the cerebellar connection in energetic dysfunctions linked to defective ATP production. We suggest that screening for mitochondrial DNA variants should be included in the diagnostic workup of selected ataxic patients, even in cases of negative family history, especially in complicated cases with widespread central and peripheral nervous system involvement.

### Supplementary Information

Supplementary Material 1.

## Data Availability

The datasets used and/or analysed during the current study are available from the corresponding author on reasonable request.
